# Babesiosis: Appreciating the Pathophysiology and Diverse Sequela of the Infection

**DOI:** 10.7759/cureus.11085

**Published:** 2020-10-21

**Authors:** Juan Fernando Ortiz, Paul W Millhouse, Álvaro Morillo Cox, Leticia Campoverde, Arveen Kaur, Martín Wirth, Adam Atoot

**Affiliations:** 1 Neurology, California Institute of Behavioral Neurosciences & Psychology, Fairfield, USA; 2 General Practice, Drexel University College of Medicine, Philadelphia, USA; 3 Medicine, Universidad San Francisco de Quito, Quito, ECU; 4 Oncology, Beth Israel Deaconess Medical Center, Boston, USA; 5 Medicine, California Institute of Behavioral Neurosciences & Psychology, Fairfield, USA; 6 Neurology, Universidad San Francisco de Quito, Quito, ECU; 7 Internal Medicine, Palisades Medical Center, North Bergen, USA

**Keywords:** babesiosis, anaplasmosis, lyme's disease

## Abstract

Babesiosis is a blood-borne disease found mainly in the United States caused by a parasitic piroplasm. While most infections are mild to moderate in immunocompetent hosts, life-threatening complications can occur in those with significant comorbidities like congestive heart failure (CHF) or chronic obstructive pulmonary disease (COPD). There is sparse literature discussing the complications of *Babesia microti* infection or the pathophysiology and management thereof. A literature review was conducted to consolidate the current knowledge about the disease, pathophysiology, and proposed management of all potential complications based on risk factors and other clinical information. A MeSH cross-references strategy was employed in PubMed using the search terms “babesia” and “babesiosis” and the established associated conditions, and the search expanded to increase capture. Only papers written in the English language and discussing human subjects in the North American patient population were included. The initial search yielded 315 papers and, after applying the inclusion/exclusion criteria, a final number of 18 was reviewed. The various complications and pathophysiology thereof are then discussed according to organ system. *Babesia* is a subversive parasite associated with a variety of conditions. We hope a better appreciation of all potential presentations and complications will help clinicians manage this increasingly common zoonosis and reduce adverse effects. More research is recommended into the pathophysiology and prevention of complications following this and other tick-borne illnesses.

## Introduction and background

Babesiosis is a malaria-like infectious disease endemic to the Northeast and Upper Midwest regions of the United States and increasingly on the Pacific Coast. The agent typically associated with infection in this area is *Babesia microti*, a parasitic blood-borne piroplasm that infects erythrocytes [1]. This protozoan accounted for the greatest number of fatalities associated with transfusion-transmitted microbial infections reported to the Food and Drug Administration (FDA) over the five-year reporting period ending 2014 (four out of 15 or 27%) [2]. From 2011 to 2015, the United States Centers for Disease Control and Prevention (CDC) reported a total of 7,612 cases in the following seven states: Wisconsin, Rhode Island, New York, New Jersey, Minnesota, Massachusetts, and Connecticut [1,3]. 

The illness can be broadly categorized into asymptomatic, mild to moderate, and severe cases. Common symptoms include fever (as high as 41°C or 106°F), nonproductive cough, arthralgia, anorexia, nausea, headaches, and fatigue. Physical examination findings depend on disease severity and comprise hepatosplenomegaly, retinal hemorrhage, and pharyngeal erythema. Typical laboratory findings may be consistent with hemolytic anemia and thrombocytopenia [4]. Diagnosis is made by microscopic visualization of *Babesia* parasites in red blood cells on a blood smear [5].

The primary disease vector for the protozoan is *Ixodes scapularis*, commonly known as the blacklegged or deer tick [1]. This tick is endemic to the mid-Atlantic, north-central, and northeastern United States, while the related western blacklegged tick (*Ixodes pacificus*) is the disease vector on the West Coast of the US [6]. This same organism can be responsible for the transmission of three known infectious diseases: *Babesia microti* (babesiosis), *Borrelia burgdorferi* (Lyme disease), and *Anaplasma phagocytophilum* (granulocytic anaplasmosis). Initially babesiosis may be suspected clinically based on symptoms and history of tick exposure, and laboratory workup is focused on differentiating these three distinct entities. Table 1 summarizes the primary clinical features, diagnostic methods, and typical management strategies for these assorted infections [7-9].

**Table 1:** Clinical features, diagnosis, and treatment of babesiosis, anaplasmosis and Lyme disease. PCR: polymerase chain reaction, Ig: immunoglobulin, IFA: indirect fluorescent antibody

Infection	Clinical Features	Diagnosis	Management
Babesiosis	fatigue and malaise fever up to 40.9°C (105.6°F) chills, sweats, anorexia, headache, myalgia, nausea, cough, and arthralgia Physical examination: Hepatosplenomegaly, pharyngeal erythema, jaundice, and retinopathy with splinter hemorrhages and retinal infarcts	Positive Giemsa or Wright staining of thin blood smear or Positive PCR antibody assay A history of tick exposure is useful but may be absent because the bite often is unnoticed	Mild to moderate: atovaquone plus azithromycin for 7 to 10 days. Severe disease: Clindamycin plus quinine for at least 6 weeks including 2 weeks after babesia parasites are no longer detected on blood smears
Lyme Disease	Localized stage: erythema migrans, flu-like symptoms, lymphadenopathy Disseminated stage: multiple secondary annular rashes, Flu-like symptoms, lymphadenopathy Rheumatologic manifestations: transient, migratory arthritis. single or multiple joint effusion, bone, muscle, bursae pain Cardiac manifestations: conduction abnormalities, myocarditis, pericarditis Neurologic manifestations; Bell’s palsy, meningitis, encephalitis.	If positive enzyme immunoassay or immunofluorescent essay and signs and < 30 days of symptoms: positive IgM and IgG Western Blot	Adults: doxycycline (10-21 days), amoxicillin or cefuroxime (14-21 days) Children: amoxicillin (14-21 days) Neurological or cardiac forms: intravenous ceftriaxone or penicillin.
Anaplasmosis	Early illness: Fever, chills, Severe headaches, Myalgias, nausea, vomit, diarrhea, loss of appetite Severe illness: Respiratory failure, Bleeding problems, Organ failure, Death Laboratory findings: leukopenia, thombocytopenia	Based on clinical signs and symptoms, and can be confirmed by: IFA or PCR testing, or characteristic intraleukocytic morulae on peripheral blood.	Doxycycline in all ages

While the acute presentation, diagnosis, and management of babesiosis are widely discussed in the literature, the chronic manifestations and associated conditions are less described. Sparse literature exists discussing epidemiology or the pathophysiology of each potential complication. In order to consolidate the knowledge of babesiosis and gather greater insight into the sequela of this rare but increasingly prevalent disorder, a literature review was conducted. Our objective was to increase appreciation for the wide variety of presentations and complications associated with this entity. Risk factors and complications as well as the pathophysiology of the complications are discussed. We hope this may help clinicians recognize and manage associated illnesses and ultimately prevent a chronic clinical picture.

## Review

Methods

A PubMed Medical Subject Headings (MeSH) search was conducted using major topics “babesia” and “babesiosis” along with any terms from a list of known associated conditions. An iterative strategy was employed wherein additional complications or comorbidities found in these results were fed back into the query, ultimately yielding the final strategy. The Yale MeSH Analyzer [10] was also used to expand the number of search terms and broaden the results. The list of references was also screened for papers that may be of interest to this study.

Following this iterative process, the results were filtered by human subjects and papers written in the English language. If available, the abstracts were then reviewed according to the inclusion and exclusion criteria. Only journal articles discussing patients from the United States were included while studies involving microbiological or animal research, those without available abstracts or free full-text were excluded. The resulting manuscripts were then reviewed to expand the list of complications.

Results

The final MeSH search yielded a database consisting of 315 articles. After reviewing the abstracts and filtering this was limited to 38 manuscripts. Upon reviewing the full texts and applying the inclusion and exclusion criteria a further 22 were excluded, resulting in a dataset of 17 articles. Table 2 outlines the MeSH search strategy and resulting articles. We have also provided a list in Table 3 of all the comorbidities, complications, and other conditions associated with babesiosis discovered in the literature.

**Table 2:** Results of Medical Subject Headings (MeSH) search, filters and criteria

Search results	315
English language	312
Human subjects	189
Abstract available	124
Free full text	38
Inclusion / exclusion	18

**Table 3:** Associated conditions of babesiosis

Cardiac	Hematologic
Congestive heart failure (CHF)	Disseminated intravascular coagulation (DIC)
Coronary artery disease (CAD)	Ehrlichiosis
Myocarditis	Granulomatous anaplasmosis
	Hemolytic anemia
Pulmonary	Hemophagocytic lymphohistiocytosis (HLH)
Acute lung injury	Leukopenia
Acute respiratory distress syndrome (ARDS)	Lyme disease
Chronic obstructive pulmonary disease (COPD)	Lymphopenia
Non-cardiogenic pulmonary edema (NCPE)	Neutropenia
	Pancytopenia
Hepatorenal, other	Reactive hemophagocytosis
Hepatitis	Splenic infarction
Renal failure	Splenic rupture
Multiorgan failure	Thrombocytopenia
Death	Warm-antibody hemolytic anemia (WAHA)

Discussion

There are many effects of babesiosis, and the presentation and clinical course can vary widely. The associated conditions can be broadly categorized into cardiac, pulmonary, renal, and hematologic, with the latter comprising the largest group. While conditions such as acute respiratory distress syndrome (ARDS), congestive heart failure (CHF), disseminated intravascular coagulation (DIC), hemolytic anemia, splenic rupture, and renal failure are frequently cited complications, the incidence varies, and many times what is thought to be sequela may be an exacerbation of an underlying disease process.

In addition, this review discovered the following less commonly reported sequela or associated conditions: acute lung injury, pulmonary edema, warm autoimmune hemolytic anemia (WAHA) [11], thrombocytopenia [12], reactive hemophagocytosis or hemophagocytic lymphohistiocytosis (HLH) [13], hepatitis [14-16], hepatomegaly [14], splenomegaly [14], pancytopenia [17], septic shock [18], multiorgan failure, and even death [12-18].

Cardiac

In a study by White et al. involving 139 cases hospitalized for babesiosis, 10.9% developed CHF. This paper also discussed prognostic factors of disease severity. On univariate analysis, prior cardiac abnormalities, presence of a heart murmur, previous splenectomy, parasitemia greater than 4%, high alkaline phosphatase levels, and elevated white blood cell count were found to be statistically significant predictors of serious disease [19].

In another series describing 34 consecutive patients hospitalized for severe babesiosis, CHF was listed as a comorbidity for three (8.8%, p=0.04). Other conditions more common with *Babesia* infection versus the control cohort included splenectomy (32.3%), coronary artery disease (23.5%), and chronic obstructive pulmonary disease (COPD) (17.6%) (all statistically significant). All of the patients with associated cardiac diseases were older and male, with the youngest being 66 years old. Again, pathophysiological mechanisms were not postulated. Based on these reports it seems possible that in some cases cardiac manifestations are revealed or exacerbated by the infection and the ensuing complications, such as pulmonary edema, rather than directly caused by *Babesia* spp. [20]. Incidentally, despite the common finding of babesiosis-associated CHF in textbooks and review articles, our MeSH search for babesia terms combined with heart failure returned zero results.

There is also a recent report of babesiosis-associated myocarditis. In a paper by Kandalaft et al., an 83-year-old immunocompetent male (again with coronary artery disease) was diagnosed with myocarditis due to a massive parasite load. The authors recommended transfusion when parasite loads exceed 10% to remove infected red blood cells from circulation [21].

Pulmonary

Respiratory manifestations are rare in babesiosis, but often noted as sequela especially for hospitalized patients. Non-cardiogenic pulmonary edema (NCPE) is a frequent manifestation and is not believed to be related to splenic function or the level of parasitology. NCPE is characterized by acute pulmonary edema without pleural effusions or cardiomegaly and typically resolves rapidly with supportive management.

ARDS is a condition characterized by excessive inflammation and pulmonary edema in response to an insult to the alveolar capillary membrane. In a recently reported series, this life-threatening condition was observed in 8 to 21% of all hospitalized patients with babesiosis. The pathophysiology of respiratory distress is believed to involve diffuse alveolar inflammation secondary to release of cytokines (cytokine storm), leading to increased pulmonary permeability and edema [22]. Animal studies have also shown increased levels of tumor necrosis factor-alpha (TNFα) and interferon-gamma (IFNγ) during infection [23]. Of note, ARDS apparently seems to follow initiation of anti-parasitic therapy [22]. This suggests the lung injury may be secondary to inflammatory mediators released by dying organisms. Other contributing mechanisms involve increased levels of complement, micro-emboli, and onset of disseminated intravascular coagulation [22]. In one study, risk factors for ARDS found in association with *Babesia* included: advanced age, immunosuppression, and previous splenectomy [22]. Patients with moderate to severe disease who begin treatment should be monitored with arterial blood gases, chest radiographs, and possibly mechanical ventilation in order to safeguard the respiratory system.

Renal

Renal failure has been described as a severe complication in a small percentage of cases following *Babesia* infection. In 1998, a case report of 134 patients reported that only six patients (4.3%) developed renal failure [19]. In 2001, Hatcher et al. reported a 5.9% (two out of 34) rate of renal insufficiency in a series of cases of *Babesia*, one of which required dialysis [20]. The renal involvement in babesiosis has been attributed to acute tubular necrosis from hemodynamic instability, sepsis, and tubular injury caused by heme [24].

Numerous models have identified numerous kidney lesions associated with babesiosis. Some of the findings are renal infarcts associated with polymorphonuclears and macrophages infiltration, acute tubular necrosis with marked peritubular macrophages glomerular hypertrophy, and mesangial expansion caused by the deposition. Immune aspects such as proliferative glomerulonephritis with immunoglobulin G (IgG) and C3 deposition without tubulointerstitial changes have also been described. Renal disturbances noted in mammals infected with *Babesia* include mesangioproliferative glomerulonephritis, acute tubular injury, active interstitial inflammation with lymphocytes and macrophages, and extensive tubular necrosis with interstitial edema without a significant inflammatory infiltrate [25].

Management of *Babesia*-induced renal failure depends on concomitant conditions and severity but typically involves a conventional approach. Hemodialysis is used in severe cases, although some authors recommend the use of glucocorticoids given the immunological component [24].

Hematologic

Disseminated intravascular coagulation (DIC) is a disease involving systemic activation of the coagulation cascade, generating thrombosis and consuming the coagulation factors [26]. In the case series reported by Hatcher et al., six of 34 (17.6%) hospitalized patients with *Babesia* infection were later diagnosed with DIC. A report by Hatcher et al. suggested that age parasitemia level of ≥10% may be associated with development of DIC although this was not statistically significant (p=0.08). Only severe anemia (defined as hemoglobin ≤10) reached statistical significance (p=0.01). While other risk factors were investigated in relation to development of DIC, such as age, previous splenectomy, and association with Lyme disease, none were close to significance [20]. The pathophysiology of DIC is related to increased levels of cytokines. Rising levels of parasitemia in babesiosis and the resulting hematologic breakdown increases the cytokine levels, which leads to a state of hypercoagulation and hypo-fibrinolysis, and ultimately multiple thrombus formation [26].

The prevalence of anemia in a case series of hospitalized patients was 17.4%, while the prevalence of thrombocytopenia varied by disease severity from 19% to 41% [19]. Hypersplenism results in increased platelet sequestration and destruction by splenic macrophages. There are also reports of immune-mediated destruction of platelets and other blood products. While the main mechanism of action involves red blood cell lysis by the parasites, oxidative damage of the erythrocytes and autoimmune hemolysis also occurs. The anemia typically resolves with antimicrobial treatment and parasite clearance.

In contrast to the more typical non-immune hemolytic anemia [27], WAHA is the most common type of autoimmune anemia and occurs at body temperature. In a report by Woolley et al. WAHA developed in 7% of patients with babesiosis during the study period. All six patients were asplenic (p<0.001) and positive for direct IgG antiglobulin [11].

Management for WAHA required immunosuppressive treatment for the majority of cases. The more typical hemolytic anemia or thrombocytopenia can be followed for disease progression with remission of parasitology, and exchange transfusions can be used if necessary. Removing or embolizing the spleen may be required for severe cases [11].

Finally, HLH is a severe systemic inflammatory problem that can sometimes occur due to strong activation of the immune system during infection. This syndrome has been reported in four cases since 1986 and is believed to be due to an over function of macrophages and T cells, and lack of activity of natural killer (NK) cells [13].

Splenic rupture is a rare complication of babesiosis infection. A case series by Patel et al. describing 84 patients hospitalized for babesiosis found seven with splenic rupture. It was concluded that splenic rupture occurs in around 1% of patients with babesiosis [28]. Results from two other case series demonstrated that the vast majority of patients were male (18 out of 19 combined) [28,29]. While other disease complications may be related to the level of parasitemia, splenic rupture does not appear to be and can be present in patients with mild, uncomplicated cases [28,29].

The following mechanisms of disease progression have been proposed leading to splenic rupture: 1) Since *Babesia* is an intra-erythrocytic organism, the spleen becomes enlarged and hyper-functional, degrading the abnormal erythrocytes. 2) Degradation of spleen parenchyma as opposed to elevation of intracapsular pressure (only mild splenomegaly found in most cases). 3) Small splenic infarctions producing focal necrosis and eventually rupture. 4) Response to infection and demarginization of leukocytes increases acute phase reactants and markers of inflammation such as TNF-α, IFN-γ, and interleukin 10 (IL-10); the end result of this pathway is endothelial damage and a hypercoagulable state. The endothelial damage and microthrombus formations lead to splenic infarction and rupture [28-30].

The management of splenic rupture depends largely on hemodynamic stability; stable patients will tolerate splenectomy while sicker patients may benefit from splenic embolization. It is important to note that removal of the spleen could worsen ongoing infection and increase susceptibility to encapsulated organisms, and proper vaccination and prophylaxis is recommended [29].

Multiorgan

*Babesia* seems to spare the liver in general but hepatomegaly and transiently elevated transaminase levels have been reported [14]. Nassar et al. reported a report about an elderly gentleman who presented with fulminant liver failure found to be due to babesiosis. In this case it was presumed that the biliary tree was involved in the ongoing sepsis until *Babesia* was discovered on peripheral smear [16]. Curiously, there is also a report of a patient whose chronic hepatitis C resolved after co-infection with *Babesia*, *Borrelia burgdorferi*, and *Ehrlichia* (human granulocytic ehrlichiosis) [15]. There was also only one report in which *Babesia* was involved in septic shock, with other etiologies present [12]. Similarly, the cases involving multiorgan failure involved multiple comorbidities and complicated hospital courses. These are likely extraordinary cases that do not fit the general pattern of *Babesia*. Nonetheless babesiosis should be considered in the differential diagnosis in cases involving shock or acute liver failure with no other explanation, especially in endemic areas, as misdiagnosis can lead to treatment delays and increased mortality [16].

## Conclusions

*Babesia microti* is a subversive organism associated with a wide variety of presentations and complications. In general, *Babesia* affects the following organ systems: cardiac, pulmonary, renal, and hematologic. Respiratory problems are commonly found in hospitalized patients for babesiosis, and typically develop after the initiation of therapy. The pathophysiology of lung injury is believed to be related to increased levels of inflammatory markers, as well as release of complement, micro-emboli, and disseminated intravascular coagulation. Patients with moderate to severe disease should be monitored with arterial blood gas and chest radiographs, with mechanical ventilation on standby, to diminish the incidence of severe pulmonary edema. Congestive heart failure is commonly associated with babesiosis in several series and hardly mentioned in the other reports. Preexisting cardiac problems may be unmasked or exacerbated by infection. Splenic rupture is a rare complication that does not appear to be related to the level of parasitemia. The mechanisms involved in splenic rupture are multiple: 1) degradation of spleen parenchyma, 2) splenic infractions, 3) increased levels of TNF-α, IFN-γ, and IL-10, 4) splenic hypertrophy due to removal of the infected erythrocytes. Anemia is also quite common, possibly related to increased hemolysis, immune-mediated destruction of blood products, and splenic sequestration. Additional complications are rarely reported and likely follow a similar pathway as involved in the more common disorders. More research is recommended into the pathophysiology and prevention of complications following babesiosis and other tick-borne illnesses.
